# From the baker to the bedside: yeast models of Parkinson's disease

**DOI:** 10.15698/mic2015.08.219

**Published:** 2015-07-27

**Authors:** Regina Menezes, Sandra Tenreiro, Diana Macedo, Cláudia N. Santos, Tiago F. Outeiro

**Affiliations:** 1 Instituto de Biologia Experimental e Tecnológica, Apartado 12, Oeiras 2781-901, Portugal.; 2 Instituto de Tecnologia Química e Biológica António Xavier, Av. da República, 2780-157 Oeiras, Universidade Nova de Lisboa, Portugal.; 3 Instituto de Medicina Molecular, Av. Prof. Egas Moniz, Lisboa 1649-028, Portugal.; 4 Instituto de Fisiologia, Faculdade de Medicina da Universidade de Lisboa, Lisboa 1649-028, Portugal.; 5 CEDOC - Chronic Diseases Research Center, Faculdade de Ciências Médicas, Universidade Nova de Lisboa, Campo dos Mártires da Pátria, 130, Lisboa 1169-056, Portugal.; 6 Department of NeuroDegeneration and Restorative Research, University Medical Center Göttingen, Waldweg 33, Göttingen 37073, Germany.

**Keywords:** protein misfolding, neurodegeneration, alpha-synuclein, Parkinson’s disease, synucleinopathies

## Abstract

The baker’s yeast *Saccharomyces cerevisiae* has been extensively explored for our understanding of fundamental cell biology processes highly conserved in the eukaryotic kingdom. In this context, they have proven invaluable in the study of complex mechanisms such as those involved in a variety of human disorders. Here, we first provide a brief historical perspective on the emergence of yeast as an experimental model and on how the field evolved to exploit the potential of the model for tackling the intricacies of various human diseases. In particular, we focus on existing yeast models of the molecular underpinnings of Parkinson’s disease (PD), focusing primarily on the central role of protein quality control systems. Finally, we compile and discuss the major discoveries derived from these studies, highlighting their far-reaching impact on the elucidation of PD-associated mechanisms as well as in the identification of candidate therapeutic targets and compounds with therapeutic potential.

## INTRODUCTION

The unicellular eukaryote *Saccharomyces cerevisiae* has been extensively used as an industrial microorganism. The first records indicating its use in fermentation processes to produce alcoholic beverages and to leaven bread date back to ancient Egypt, over 5,000 years ago 1,2. Ever since, *S. cerevisiae* has been used for making bread, being therefore also frequently referred as baker’s yeast.

The first uses of *S. cerevisiae* as an experimental model organism date back to the mid-thirties of the 20^th^ century 3, and its consequent establishment as a robust model system in diverse areas of biology was largely fueled by its unique features. They include short generation time, easy handling which is further simplified by its nonpathogenic nature, inexpensive culture conditions, and, most importantly, its amenability for genetic manipulation. Being very versatile for biological and genetic studies, these attributes placed yeast at the forefront for the development of countless genetic tools to address major biological issues. Hence, for almost a century, *S. cerevisiae* has served as a remarkable experimental model for several seminal discoveries in biology, revealing important aspects of microbiology and biochemistry. For example, studies using yeast have contributed to the elucidation of fundamental cellular mechanisms involved in DNA replication, recombination and repair 4, in RNA metabolism 5, and in cell division and cell cycle progression 6.

The discovery of the high-degree of evolutionary conservation of disease genes and of fundamental biological processes among eukaryotes, combined with the power of yeast genetics, has brought *S. cerevisiae *from the baker to the bedside, as a model organism with an unprecedented potential to decipher the intricacies of devastating human pathologies such as Parkinson’s disease (PD), as well as to help in the identification of molecular targets and lead molecules with therapeutic potential.

In this review, we summarize the impact of yeast models on the current knowledge and understanding of the molecular underpinnings of PD. We first discuss the most relevant findings in the yeast field and the extent to which they have paved the way for the use of *S. cerevisiae* in biomedical research. We then briefly review the main aspects of PD, emphasizing the molecular players and pathways governing disease pathology. Finally, we cover the most important yeast models generated thus far, and discuss their contribution to the elucidation of PD-related mechanisms, as well as to the identification of molecular targets and compounds with therapeutic potential.

## THE POWER OF YEAST GENETICS 

The peculiar life cycle of *S. cerevisiae* constitutes, in itself, an invitation for performing genetic studies. In the wild, it can be found in both haploid and diploid forms that reproduce vegetatively, by budding. In nutrient-poor environments, a condition easily mimicked experimentally, diploid cells undergo meiosis and sporulate, yielding a progeny of four haploid cells. Hence, under controlled laboratory conditions, sporulation of a particular diploid cell allows the generation of different combinations of genotypes with desired genetic traits. Additionally, the life cycle of budding yeast also greatly simplifies the study of lethal mutations in heterozygous diploids as well as recessive mutations in haploid cells 7,8.

Yeast research has definitely won a place in history after the demonstration that yeast strains with a mutation in *LEU2* locus, therefore unable to grow in media depleted of the amino acid leucine, can be transformed with a chimeric ColE1 plasmid encoding the wild type (WT) yeast *LEU2* gene, and that this sequence can integrate into the yeast chromosome restoring leucine prototrophy 9. The discovery of the amenability of yeast cells for transformation opened new avenues for manipulation of yeast genome, allowing insertion or deletion of genes to generate recombinant strains. The high efficiency of the transformation process, aided by a very effective homologous recombination system, has provided yeast geneticists a tremendous flexibility in experimental design, which is currently incremented by the availability of a large collection of recombination-based Gateway vectors 7,10,11,12.

*S. cerevisiae* was also the host organism for the development of pioneering approaches to investigate the interaction between biomolecules. Taking advantage of the bi-modular nature of the yeast transcription factor Gal4, researchers generated a novel genetic system to study protein-protein interactions in which two known proteins are separately fused to the DNA-binding and transcriptional activation domains of Gal4 13. The principle of the methodology relies on the premise that the interaction between proteins reconstitutes a functional Gal4, which in turn activates expression of reporter genes. After its original description, a number of “variations on the theme” has been described to allow the study of DNA-protein (one-hybrid), RNA-protein (RNA-based three-hybrid) and small molecule-protein interactions (ligand-based three-hybrid), as well as to identify mutations, peptides or small molecules that dissociate macromolecular interactions - the reverse *n*-hybrid systems 14. Additionally, a split-ubiquitin membrane-based two-hybrid assay (also referred as membrane yeast two-hybrid - MYTH) was designed to overcome the limitation of the original system on the assessment of protein interactions forced to occur within the nucleus, for membrane-embedded proteins 15,16. The fact that novel hits for a target protein, from both yeast and other organisms, can be identified in the screening of libraries, without any prior bias or knowledge of their identity, is the most powerful application of these techniques.

Another tool allowing the study of protein interactions *in vivo* is the bimolecular fluorescence complementation (BiFC). This method, originally developed in mammalian cells 17,18, has been efficiently used in yeast to visualize protein interactions with minimal perturbation of the normal cellular environment 19,20. It is based on the principle that two fragments of a fluorescent protein are each fused to target proteins. The reassembly of these non-fluorescent fragments into a fluorescent complex is mediated by the interaction between the target proteins, thereby constituting a powerful tool to resolve spatial and temporal aspects of many molecular interactions 21.

The genetic features highlighted have distinguished yeast as a versatile model organism. As such, *S. cerevisiae* was the first eukaryotic organism to have its genome fully sequenced 22. Thus, the completion of the yeast genome in 1996 represented a landmark achievement in the history of eukaryotic biology. This has been providing, in the course of the last two decades, a wealth of information allowing the development of several biological resources such as the yeast gene deletion strains 23, the tetracycline (tet)-repressible 24 and heat-inducible shutoff set of strains 25 to generate conditional mutants of essential genes, the GFP- 26 and TAP-tagged 27 collection of strains, collections of other protein tags, and collections engineered for protein overexpression 28,29,30. A compendium of existing tools and resources available for the yeast research community is provided by Tenreiro and Outeiro 31 and by Duina and coauthors 7.

As the most facile eukaryotic model organism for experimental biology, yeasts were placed at the forefront for the establishment of new research fields: (1) functional genomics, in particular transcriptomics (DNA microarrays and RNA-sequencing), proteomics, metabolomics, interactomics and locasomics (protein subcellular localization) 11, employing the resources detailed above in a high-throughput automated setup; and (2) systems biology, to scrutinize the huge amount of data generated towards building a comprehensive model of eukaryotic cell functioning 7,8,32. Compilations of genetic and biological data from these analyses, including information regarding predicted orthologues in humans, are easily accessible at the comprehensively annotated *Saccharomyces* Genome Database online resource (http://www.yeastgenome.org) 33 and other public databases 7,31,34.

The power of yeast genetics has fueled all the achievements discussed, rendering *S. cerevisiae* as the best-understood eukaryotic organism. A surprising, but delightful finding, emerging from the cumulative knowledge on yeast genomics and biology, was the unpredictable gene homology and functional conservation of key fundamental cellular processes between yeasts and higher eukaryotes 4,5,8.

Importantly, the *S. cerevisiae* genome encodes nearly 1000 genes which are members of orthologous gene families related to human disease, representing about 20% of the total yeast genes 35. The mammalian orthologues of most of these genes are functional in yeast and complement the respective yeast deletion mutant. In line with the evolutionary conservation of disease genes in eukaryotes, it has been extensively shown that several disease-associated cellular pathways are also highly conserved from yeast to humans 11,34,36, enabling the modeling of specific disease aspects in this model organism. Protein quality control systems 37, vesicular trafficking and secretion 38, autophagic pathways 39, the unfolded protein response 40, and mitochondrial biogenesis and metabolism 41 are among the conserved cellular mechanisms, allowing the study of fundamental mechanisms associated with neurodegenerative diseases, such as PD, in yeast cell models 11.

Modeling particular molecular aspects of human diseases in yeast models can be achieved using distinct strategies, depending on the presence or absence, of disease-genes orthologues in the yeast genome 42. If the genes of interest have yeast counterparts, a unique opportunity to directly study their function is offered, either through their deletion or overexpression. In a more physiological context, human wild type (WT) and mutant alleles of favorite genes can be heterologously expressed in the respective yeast mutant backgrounds, since they are capable of replacing the function of the endogenous yeast gene product. Otherwise, if the disease-associated genes do not have a yeast orthologue, a functional analysis can still be conveniently performed via heterologous expression in WT strains 31,43 to provide paradigms of their function on cellular physiology and metabolism. Insights into the function of most PD-associated genes have been obtained using the latter strategy. However, the study of endogenous yeast proteins has been also providing clues on the role of key PD players, as discussed in detail bellow.

Once comprehensively validated as reliable experimental model systems to recapitulate specific aspects of human diseases, “humanized” yeasts constitute powerful toolboxes for high-throughput screenings of genes that may constitute therapeutic targets, and as robust primary drug-screening platforms to filter for cytoprotective compounds 31,44.

Despite several singularities and idiosyncrasies of yeast cells, their simplicity can be turned from a limitation into an asset, by virtue of all the attributes mentioned above. Thus, *S. cerevisiae* is uniquely suited for the task of assisting our understating of the cellular mechanisms underlying human diseases as well as in the search for novel molecular targets and compounds prone for therapeutic intervention.

## PARKINSON'S DISEASE AND THE KEY GENETIC PLAYERS 

PD, described by James Parkinson in 1817 45 and then further refined by Jean-Martin Charcot 46, is one of the most common neurodegenerative disorders. Currently, it is estimated that there are 4 million diagnosed PD patients worldwide. However, it is estimated that 7 to 10 million people live with this devastating chronic disease (data from the Parkinson’s Disease Foundation). The typical motor symptoms of PD include tremor at rest, bradykinesia, stiffness, and postural instability 47. These symptoms are caused by the progressive degeneration of nigrostriatal dopaminergic neurons from the *substantia nigra pars compacta* of the brain. Nevertheless, PD is currently considered as a whole-brain disorder, affecting multiple brain areas and presenting a broad variety of symptoms 48. The histopathological hallmark of PD, and other synucleinopathies, is the appearance of proteinaceous intraneuronal cytoplasmic inclusions termed as Lewy bodies (LB) and Lewy neurites 49. These insoluble aggregates are predominantly composed of alpha-synuclein (aSyn) 49, a protein encoded by the *SNCA* gene, and are decorated with components of protein quality control systems such as molecular chaperones, and proteasomal and lysosomal subunits 50.

The *SNCA* gene was the first genetic locus to be associated with familial forms of PD, and was later also implicated in sporadic cases of the disease 51. It encodes aSyn, a protein whose function remains unclear, but that has been proposed to be linked to diverse functions ranging from transcriptional regulation 52,53,54,55,56,57, mitochondrial homeostasis 58 and vesicle trafficking 59,60,61, possibly regulating dopamine neurotransmission, synaptic function and synaptic plasticity 62,63,64,65.

Genetic alterations in the *SNCA* gene linked to PD include duplication or triplication of the *SNCA* locus 66,67,68, as well as missense mutations A30P 69, E46K 70, H50Q 71,72, G51D 73, A53T 51,74,75, and A53E 76, causing autosomal dominant forms of the disease. The precise effect of each of these mutations is unclear, but they seem to affect the interaction of aSyn with membranes 77,78,79,80,81,82, and to alter the propensity of the protein to aggregate, at least *in vitro* 81,83,84,85,86,87,88,89.

As a common genetic determinant of both idiopathic and inherited forms of PD, and the major component of LB, much of the work in the PD field converges on aSyn (also called PARK1). However, several genes linked to heritable, monogenic PD, have been described. These include the leucine-rich repeat kinase 2 LRRK2 (PARK8), the E3 ubiquitin-ligase Parkin (PARK2), the mitochondrial PTEN-induced putative kinase 1 Pink1 (PARK6), the oxidation-sensitive chaperone DJ-1 (PARK7), and the lysosomal ATPase ATP13A2 (PARK9) 90. Additionally, several genes are known to be associated with an increased risk of developing PD, such as the vacuolar protein sorting 35 homolog VPS35 (PARK17), the ubiquitin carboxyl-terminal esterase L1 UCH-L1 (PARK5), the translation initiation factor 4-gamma 1 EIF4G1 (PARK18), and beta-glucocerebrosidase (GBA) 90. A comprehensive list of PD genetic risk factors, including the six risk loci associated with proximal gene expression or DNA methylation recently identified in large-scale meta-analysis of genome-wide association studies, are available online at ‘PDGene’ (http://www.pdgene.org) 91,92.

Mutations in LRRK2, encoding a 2527-amino acid cytosolic kinase, are the most frequent genetic cause of PD 93,94. The role of this kinase has been associated with biological processes such as endocytosis of synaptic vesicles 95,96, autophagy 97, and neurite outgrowth 98. The recent discovery of LRRK2 interactions with members of the dynamin superfamily of large GTPases, by yeast two-hybrid analyses, implicates its function in the regulation of membrane dynamics relevant for endocytosis and mitochondrial morphology 99. In line with these findings, LRRK2 appears to modulate the cellular protection against oxidative insults in a mechanism that is dependent on endocytosis and mitochondrial function 100. LRRK2 was also shown to interact with aSyn 101,102, possibly mediating its phosphorylation on S129 103. The pathogenic mechanisms triggered by mutant versions of LRRK2 are still unclear 94. However, it is speculated that mutations may affect its interactions with other proteins 90, possibly also with aSyn.

Parkin homozygous mutations are the most frequent cause of juvenile PD. Parkin has been associated with various cellular pathways but special importance has been given to its role in mitochondrial quality control, where it participates in common pathways with Pink1 to regulate the formation of mitochondrial-derived vesicles and mitophagy 104,105,106,107,108,109,110. Indeed, the MVD pathway has been referred as the primary defense mechanism against mitochondrial damage. Mitophagy only plays a role once MVDs are overwhelmed. Parkin-assisted Pink1 translocation into mitochondria is associated with autophagy of damaged mitochondria 107,109, and was further supported by yeast studies on the modulation of mitochondrial degradation upon oxidative injury and chronological aging 111.

The DJ-1 protein, the most extensively studied member of the DJ-1 superfamily, is a multifunctional protein associated with numerous cellular functions including oxidative stress responses 112. The cellular mechanisms by which mutations in DJ-1 cause PD are still unclear, but DJ-1 may act as a redox-dependent chaperone preventing aSyn aggregation 113. DJ-1 overexpression confers protection against neurodegeneration in model organisms, in a mechanism that is dependent on protein-protein interactions between DJ-1 and disease proteins 114,115. This suggests that the direct interaction between DJ-1 and its targets constitutes the basis for the neuroprotective effect of DJ-1. Further insights into putative roles of members of the DJ-1 superfamily were obtained in yeast, where Hsp31-like chaperones may serve as regulators of autophagy 116.

Finally, it is important to stress that most of the genes associated with PD encode proteins whose functions appear to be related to mitochondrial function, membrane trafficking and protein quality control systems, underscoring the importance of these mechanisms in the pathophysiology of PD.

## MODELLING PD IN YEAST 

**Figure 1:**
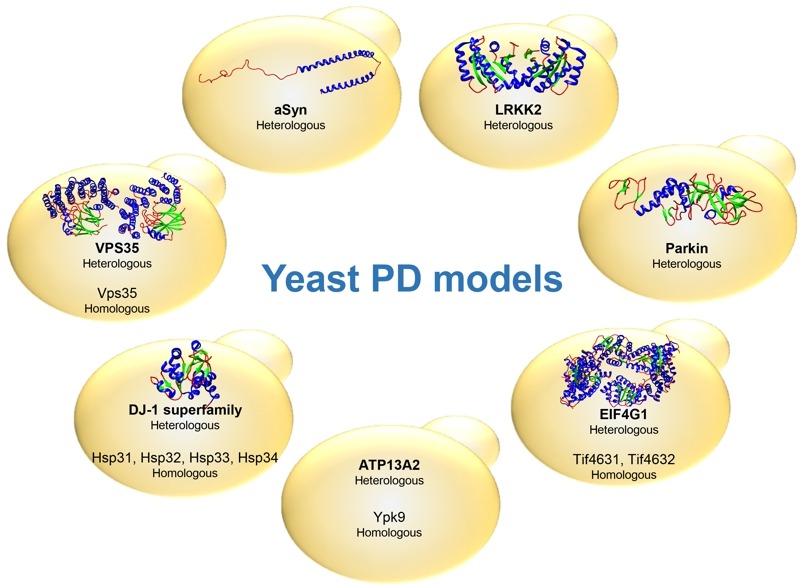
FIGURE 1: Schematic representation of yeast PD models. The PD-associated proteins are indicated as well as the type of expression (heterologous or homologous). The structure of the proteins is represented for aSyn, LRRK2, Parkin, EIF4G1, DJ-1 and VPS35. Protein Data Bank ID: 1XQ8, 2ZEJ, 4I1H, 2VSX, 4OQ4 and 2R17 respectively. The structure of ATP13A2 is still undetermined.

The identification of genes associated with Mendelian forms of PD enabled major leaps forward in our understanding of the molecular mechanisms involved in the disease. This also led to the development of various cell and animal models that are widely used. Among these, yeast models have proven extremely useful to dissect the basic molecular mechanisms associated with PD and other synucleinopathies (Table 1). These models are based either on the heterologous expression of the human genes, or by studying the function and pathological role of the yeast counterparts, when these are represented in the yeast genome (Fig. 1).

**Table 1:** Yeast models of PD.

**Human gene**	**Human protein**	**Yeast homolog**	**Yeast model**	**References**
*SNCA*	alpha-synuclein	__	heterologous expression of human aSyn	117,121
*PARK8*	LRRK2	__	heterologous expression of human LRRK2	135
*PARK7*	DJ-1	*HSP31,* *HSP32,* *HSP33* and *HSP34*	functional analysis of the yeast homologs; co-expression of DJ-1 with aSyn*;* co-expression of DJ-1 with PD associated mutations	114,116
*PARK2*	Parkin	__	heterologous expression of human Parkin	111,139
*PINK1*	Pink1	__	heterologous expression of human Pink1	111,139
*VPS35*	VPS35, or PARK17	*VPS35*	functional analysis of the yeast homolog	144
*EIF4G1*	EIF4G1	*TIF4631 *and* TIF4632*	functional analysis of the yeast homologs	144
*ATP13A2*	ATP13A2	*YPK9*	co-expression of Ypk9 with human aSyn; co-expression of Ypk9 with PD associated mutations	127,146,147,191

### aSyn

The first yeast model of PD was based on the heterologous expression of human aSyn 117. Since then, many other studies used this approach to investigate the molecular mechanisms underlying aSyn toxicity and to identify novel compounds of therapeutic interest, as described below.

The expression of aSyn in yeast results in dose-dependent cytotoxicity 117, as observed in other model systems. This is also in line with the identification of familial forms of PD associated with duplications 67 and triplications 66 of the aSyn locus. In addition, expression of aSyn in yeast also resulted in the formation of intracellular inclusions. The nature of these inclusions was matter of debate, as it was observed that aSyn leads to the accumulation of vesicles 118,119,120, raising doubts on whether the inclusions observed were indeed aggregated aSyn. However, amyloid-like aggregates of aSyn seem to be also formed in yeast cells, as some inclusions are positively stained by thioflavin S 121 or thioflavin T 122. More recently, the formation of large oligomeric species of aSyn in yeast cells was also demonstrated using both sucrose gradients and size exclusion chromatography 123.

The various yeast models based on the heterologous expression of aSyn present different phenotypes, depending on the expression system, given that this affects the level of aSyn expression 117,124,125. This feature has been also explored according to the objectives of the studies.

The use of multicopy plasmids revealed that yeast cells reduce the average plasmid copy number in order to reduce aSyn expression and toxicity 117. To avoid this, insertions of the aSyn coding sequence in the yeast genome enabled more stable expression, and the levels of toxicity could be manipulated by varying the number of copies of the aSyn cDNA inserted in the genome 125,126. The use of a galactose-inducible promoter provided additional control for the synchronous induction of expression of aSyn, avoiding the negative pressure during routine cell manipulations.

Using these various expression systems, several genetic modifiers (enhancers and suppressors) of aSyn toxicity were identified in genetic screenings in yeast 124,125.

In other studies, yeast cells expressing different levels of aSyn, hence displaying different levels of cytotoxicity, revealed the involvement of multiple cellular pathways in the toxicity 117,119,125,126,127,128,129. In turn, this facilitated the identification of synergistic effects between different genetic suppressors of toxicity 126,127. Overall, these studies greatly expanded our understanding of the cellular pathways affected by aSyn. Among these, ER-to-Golgi trafficking appeared to be significantly disrupted 125. Also, mitochondrial stress was identified as an early signature of aSyn toxicity 126. Indeed, the aSyn expression system controlled by the yeast *MET25* promoter 121 enabled the verification that aSyn cytotoxicity requires functional mitochondria 130. Moreover, mitochondria has been pointed out as the site of enhanced ROS production in response to Pmr1-dependent Ca^2+^ overload leading to cellular death in yeast, flies and worms aSyn models 131.

Other effects of aSyn overexpression in yeast are impairment of proteasome activity 117, accumulation of cytoplasmic lipid droplets 117, ER stress 125, activation of the heat-shock response 128, mitochondrial dysfunction 126, shorter chronological life span and induction of autophagy and mitophagy (mediated by Sir2) 132, impairment of endocytosis 117, ROS production and induction of apoptosis 126,133. Recently, it was shown that apoptosis is dependent on the translocation of Endonuclease G (EndoG) from the mitochondria to the nucleus, where it mediates DNA degradation 134.

### LRRK2

The role of other genes associated with monogenic forms of PD has also been studied in yeast models. Namely, important insights into the function and the pathogenic mechanisms of LRRK2 mutations were obtained in yeast cells heterologously expressing human LRRK2 100,135. Higher levels of expression of the full-length LRRK2, based on a multicopy expression vector and on a galactose inducible promoter, resulted in the accumulation of insoluble protein but no alterations in the phenotype 100,135. However, with lower levels of LRRK2 expression, a phenotype of protection against oxidative stress is conferred by LRRK2 100. Interestingly, the protective effect of LRRK2 is lost when PD-associated mutants are used instead of the WT protein 100.

The effects of the overexpression of various functional domains of human LRRK2 were also analyzed in yeast 135. The GTPase activity of LRRK2 was found to be inversely correlated with cytotoxicity 135. This toxicity was correlated with defects in endocytic trafficking and autophagy 135. These results were further validated in mouse primary cortical neurons were the overexpression of full-length LRRK2 causes defects in both synaptic vesicle endocytosis and exocytosis 135. This study also revealed that aSyn and LRRK2 cause vesicular trafficking-associated toxicity through distinct pathways that, nevertheless, culminate in trafficking defects to the vacuole, the yeast counterpart of the lysosome 135.

### DJ-1

In yeast, there are four homologous and highly conserved genes that belong to the DJ-1 superfamily: Hsp31, Hsp32, Hsp33 and Hsp34. Recently, it was found that these proteins are required for metabolic reprogramming triggered by glucose limitation, known in yeast as diauxic-shift 116. It was also found that these DJ-1 homologs contribute to target of rapamycin complex 1 (TORC1) regulation 116, a central player in diauxic-shift reprogramming and, importantly, in autophagy 136. Both TORC1 and autophagy are dysfunctional in several pathologies including PD 137,138. Thus, this study constitutes an example of how the functional analysis of yeast homologs provides important insight into the putative functions of human genes associated with disease.

In a separate study, the human DJ-1 gene was expressed in yeast cells using a multicopy vector 114. It was found that DJ-1 interacts with aSyn and that PD-associated mutations impair this interaction 114. Interestingly, it was found that DJ-1 and the yeast homologs attenuate aSyn toxicity in yeast 114. Thus, these findings suggest that the physical interaction between DJ-1 and aSyn might represent a neuroprotective mechanism that is disrupted by familial mutations in DJ-1, thereby contributing to PD 114.

### Parkin and Pink1

Recently, human Parkin was expressed in yeast and, although the protein was found to be cytosolic, it was translocated to mitochondria under oxidative stress or aging, accelerating mitochondrial degradation 111. Moreover, Parkin promotes chronological longevity and oxidative stress resistance through a mitochondria-dependent pathway 111. In the same study, Pink1 was also expressed in yeast and was found to promote resistance to oxidative stress 111. However, co-expression of both proteins does not show a synergistic effect, suggesting the two proteins affect the same pathway independently 111.

Recently, the mechanism underlying Parkin activation by Pink1 was dissected in an elegant study where a yeast model was used 139. In particular, full activation of the E3 activity of Parkin E3, in response to mitochondrial damage, was found to occur in a two-step mechanism, involving the phosphorylation of both Parkin and ubiquitin, by Pink1 139.

### VPS35 and EIF4G1

The first indications highlighting the importance of vesicle-trafficking genes as modulators of aSyn toxicity resulted from genetic screens performed in yeast. Deletion of *VPS24*, *VPS28*, *VPS60* or *SAC2* was found to increase aSyn toxicity 124. These genes are involved in protein sorting in the late Golgi, sorting to the prevacuolar endosomes, for protein sorting, and trafficking to the vacuole, respectively. In another screen, *ENT3*, involved in protein transport between the trans-Golgi network and the vacuole, was also identified as a suppressor of aSyn toxicity using a yeast model 140. Only more recently, next-generation sequencing and genome-wide association studies implicated mutations in *VPS35* (PARK17) in familial cases of PD 141,142. Genome-wide association studies also allowed the identification of *EIF4G1* (PARK18) as a novel autosomal dominant PD gene 143. These genes are known to function in regulating vacuolar transport (*VPS35*) and transcription/translation (*EIF4G1*), and are highly conserved from yeast to humans. Yeast has a *VPS35* homolog, also called *VPS35,* and two *EIF4G1* homologs, *TIF4631* and *TIF4632*. These two genes were found to interact functionally and genetically, and to converge on aSyn, in a study that combined yeast, worms and transgenic mouse models 144. This study started with two independent genetic screens in strains deleted for *VPS35* or *TIF4631 *and enabled the identification of synthetic sick or lethal genes. The results pointed to a common pathway associated with both genes: the retromer complex function, important in the regulation of recycling, sorting, and trafficking between the endosomal and Golgi network. The impairment of a functional retromer complex results in the accumulation of protein misfolding, thereby exacerbating the accumulation and toxicity of aSyn 144. In particular, it was found that overexpression of *EIF4G1* (or the yeast homolog *TIF4631*) in cells lacking *VPS35* was highly toxic. This toxicity was found to be due to the loss of retromer function, which could be restored by WT *VPS35* but not by the PD-associated mutant D620N 144.

### ATP13A2

Mutations in the gene encoding the lysosomal P-type ATPase ATP13A2 cause Kufor-Rakeb syndrome and early-onset PD 145. Yeast has an orthologue of ATP13A2, the *YPK9 *gene*, *which encodes a vacuolar transporter with a possible role in sequestering heavy metals 146. The understanding of role of ATP13A2 in PD and how missense mutations could lead to a loss-of-function of the protein was facilitated by studying the yeast homolog, followed by validation in other model systems. Namely, *YPK9* was found to be a suppressor of aSyn toxicity in yeast 127. This protective function depends on the vacuolar localization and ATPase activity of Vps35 127, and is probably related to its role in homeostasis of manganese and other divalent heavy metal ions 146,147, which are recognized environmental risk factors for PD 148. Among the most common PD genetic players, ATP13A2 is the unique whose structure has not yet been unveiled.

## aSYN AND PROTEIN QUALITY CONTROL SYSTEMS IN YEAST 

**Figure 2:**
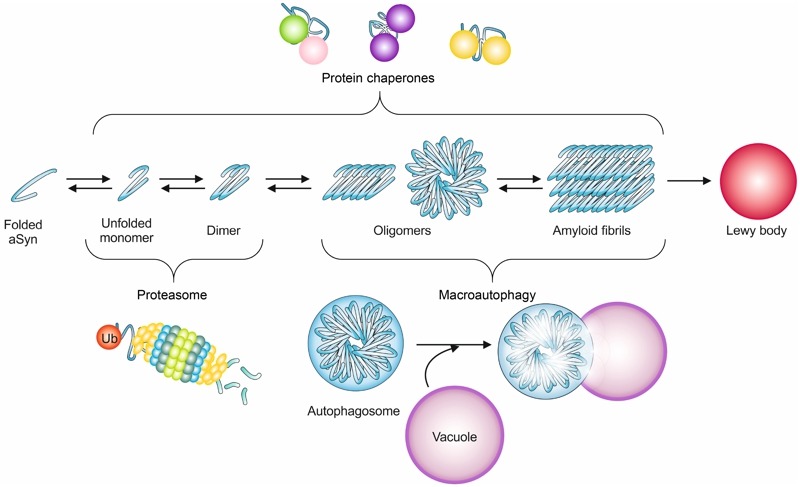
FIGURE 2: Quality control systems and aSyn aggregation. aSyn can misfold and form oligomeric species that fibrillate and deposit into larger aggregates, ultimately forming Lewy bodies. In healthy cells, the cellular quality control systems are able to maintain proteostasis, avoiding this cascade of events. The first steps of aggregation can be prevented or reversed by promoting the degradation of misfolded proteins by the ubiquitin-proteasome system, while the later ones are counteracted by degradation mediated by macroautophagy. Chaperones are thought to assist in the proper folding of aSyn at different stages of the aggregation process.

Alterations in proteostasis occur in several types of disorders 149. When the accumulation of misfolded proteins surpasses the capacity of the cell to cope with the protein load, diverse quality control mechanisms are called to action, to actively sequester, refold, and/or degrade these proteins 150,151,152,153 (Fig. 2). These cellular protein quality control mechanisms are conserved from yeast to mammalian cells 37.

Strong evidence on the involvement of the quality control systems in neurodegeneration came from studies of familial forms of PD, as mutations in several genes playing a role in these pathways are intimately associated with the disease, as mention above.

Besides those players, several molecular chaperones were also found to have an important role in PD. Namely, the involvement of molecular chaperones in aSyn yeast toxicity was evidenced by the enhanced aSyn inclusion formation observed upon deletion of individual chaperones 154. Concomitantly, pharmacological activation of the heat shock response upon treatment with geldanamycin or overexpression of the chaperones Ssa3 155, Jem1 or Hsp90 140 protected yeast cells against aSyn-induced ROS and subsequent toxicity. These data elegantly recapitulated results obtained with neuronal cell lines 156, transgenic flies 157 and mice 158,159.

The protein Gip2, an activator of the heat-shock transcription factor Hsf1, was also identified as a multicopy suppressor of aSyn toxicity, by triggering a heat-shock response in yeast 128. A screening of a mouse brain-specific cDNA library identified the mouse chaperone RPS3A as a suppressor of aSyn WT and A53T toxicity in yeast 160. Moreover, co-expression of RPS3A delayed the formation of aSyn inclusions 160. Overexpression of DJ-1, which has protein chaperone-like activity, was also described as a negative regulator of aSyn dimerization 114.

Finally, the heat shock-induced protective mechanism may involve Hsp104 and its co-chaperones, which were described to relieve cells from ER-stress 161. It was shown that Hsp104 degraded aSyn in a concentration-dependent manner and decreased aSyn fibrillation *in vitro *162,163,164. In agreement, Hsp104 antagonized aSyn aggregation and reduced dopaminergic degeneration in a rat model of PD 165. However, the studies in yeast are still scarce. It was shown that at endogenous levels the presence of Hsp104 had a deleterious effect on aSyn aggregation, and deletion of Hsp104 in yeast expressing WT or mutant aSyn resulted in lower oxidative stress, cytotoxicity, increased cell viability and rescue of endocytic defects 166. Nevertheless, another study reported that it is possible to reprogram Hsp104 to rescue aSyn proteotoxicity in yeast by mutating single residues 167. These potentiated Hsp104 variants enhanced aggregate dissolution, restored protein localization, suppressed proteotoxicity in yeast 167, and attenuate dopaminergic neurodegeneration in a *C. elegans* PD model 167.

Additional strong evidences of the relevance of the clearance pathways in PD arrive from yeast models. Namely, the expression of aSyn in yeast promotes proteasome impairment 117. This reduced proteasome activity was found to be the result of a deficient proteasome composition 168. Furthermore, the failure of the UPS (ubiquitin-proteasome system) enhances aSyn toxicity 169 170 and leads to the accumulation of inclusions 121.

Despite the clear involvement of proteasome dysfunction in PD, the degradation of aSyn inclusions is more dependent on autophagy than on proteasome function, at least in yeast 171. This is consistent with the proteasome being responsible for the degradation of soluble forms of aSyn, and suggests a complex cross-talk between the different proteolytic pathways involved in the degradation of aSyn 172 (Fig. 2).

Autophagy involves the formation of an autophagosomal vesicle that transports the misfolded and aggregated proteins to degradation, in the lysosome in higher eukaryotes, or in the vacuole in yeast. The first molecular insights into autophagy were learned from yeast 173, and as in mammalians, the process is regulated by the kinase “target of rapamycin” (TOR) pathway 174. Concomitantly Lst8, a TOR-interactor was identified as modulator of aSyn toxicity in yeast 128. Moreover, Ypk9, a vacuolar P-type ATPase that is the orthologue of ATP13A2, was identified as a suppressor of aSyn toxicity 128 and aggregation 127.

More recently, it was reported that deletion of the autophagy related genes *ATG1* or *ATG7* lead to impaired degradation of aSyn and increased toxicity 123. In agreement with the beneficial role of autophagy on aSyn toxicity, rapamycin treatment, which induces autophagy by inhibiting TOR, was reported to reduce aSyn aggregation 121. It also appears that toxic forms of aSyn lead to the impairment of autophagy and that inducing autophagy or increasing the autophagic flux is protective against aSyn toxicity in yeast 123,175.

Nevertheless, there is still controversy regarding the role of autophagy on aSyn toxicity. A study reported that rapamycin treatment increases aSyn toxicity in yeast 128. It was also shown that WT or A53T mutant aSyn were not able to enter the vacuole and promoted vacuolar fusion defects in yeast 154. Additionally, aSyn-mediated mitophagy, a specific degradation of mitochondria through autophagy, was reported to be deleterious in aged yeast cells 132. To intensify the discussion, aSyn Lewy body-like aggregates resisted degradation and impaired autophagy in mammalian cell models 132.

It is clear that the interplay between autophagy, aSyn toxicity and aggregation is still elusive. A reasonable explanation for the toxicity of aSyn mediated by autophagy induction is that the excessive activation of a dysfunctional autophagy will lead to a loss of selectivity, resulting in the trapping of functional competent proteins and organelles in autophagosomes. Ultimately, this could lead to a loss of function and cell toxicity. Thus, the beneficial or detrimental role of autophagy should be studied having in consideration its functionality and selectivity, as well as the size and nature of the aSyn aggregates.

## YEAST AS A DRUG DISCOVERY PLATFORM FOR PD 

The identification of therapeutic compounds for neurodegenerative disorders is of utmost importance. These devastating illnesses only have, in some cases, symptomatic therapies being therefore disruptive and costly for society. Thus, intense efforts are being made to understand the molecular underpinnings of neurodegenerative diseases and to identify novel therapeutic strategies. Nevertheless, given the complexity of the mechanisms leading to neurodegeneration, and the limitations in the models available, drug discovery is often slow, challenging and with limited success.

In the context of PD, drug discovery efforts focused on aSyn are complicated by the fact that it is a ”natively unstructured protein”, lacking defined secondary structure under physiological conditions 176. Thus, cell-based high-throughput phenotypic assays afford important possibilities, as they are based on relevant disease-associated phenotypes induced by aSyn expression. The readouts may include viability/toxicity, aggregation, mitochondrial function, proteasome activity, among others. Once relevant molecules and targets are identified, then it is fundamental to scrutinize the mechanism of action of the potential small molecules and candidate compounds. This is where yeast cells offer a remarkable advantage, as they enable the identification of target genes and mechanisms through diverse and complementary genetic approaches, accelerating the selection of pre-clinical candidates.

In mammalian cell systems, aSyn-associated phenotypes are often mild or inconsistent, complicating the development of reliable screening platforms 11,177. Primary rat neurons, infected with lentiviruses encoding for aSyn-expressing, have been used, but they also present technical limitations. Many of these systems are more suited for secondary validation steps, focusing on candidate genes or molecules identified in yeast, for example.

Yeast affords numerous advantages at the early stages of the drug development process, in comparison to mammalian cells and animal models. Several major drugs hit the same targets and elicit the same responses in yeast as they do in humans, including statins, methotrexate, omeprazole, tacrolimus (FK506) and bortezomib (Velcade) 177,178. In spite of the obvious limitations, such as the absence of a nervous system, and the absence of numerous gene products that are only present in mammalian cells and neurons, yeast cells are ideally suited for investigating the primordial molecular events triggering cell dysfunction and pathology. In this context, yeast cells can be regarded as living test tubes, where genetic manipulations are faster and more straightforward, with rapid growth and reduced cost, and functional similarity to higher eukaryotes.

The presence of a cell wall in yeast cells poses challenges that need to be considered in the context of drug screening efforts. However, this can be minimized by genetic manipulation of the efflux pump system or the ergosterol biosynthesis, reducing the capability of yeast cells to export drugs or by increasing the permeability of the cells, respectively.

**Figure 3:**
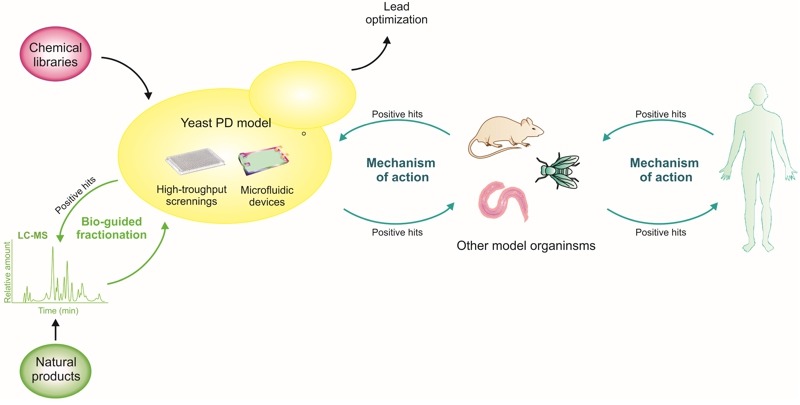
FIGURE 3: Yeast as a discovery platform for PD. The discovery of small molecules, or natural products, which are able to rescue aSyn toxicity and aggregation benefits from the combination of approaches in yeast with those in other model systems. In particular, libraries of small molecules or natural products can first be screened in yeast models of PD, and combined with LC-MS approaches in order to enable an interactive bio-guided fractionation of complex mixtures to identify candidate compounds. The development of microfluidic devices, together with yeast genetics and high-throughput approaches, are also important to elucidate the mechanisms of action of the candidate compounds. Ultimately, the mechanisms and molecules identified can be further scrutinized in an iterative process between yeast, other model systems and, ultimately, in humans.

Yeast is considered a robust primary drug-screening platform to filter for compounds with cytoprotective activity, for further complementation with assays in more physiologically relevant models. Approaches involving the sequential use of different model systems, starting with simpler cellular models and ending with more complex animal models, as schematized in Fig. 3, already resulted in the discovery of promising small molecules with therapeutic potential (described below). Recently, a yeast-to-human discovery platform for synucleinopathies was established, where genes and small molecules identified in yeast were validated in PD-patient derived neurons. Subsequently, yeast cells were again used for clarification of the mechanism of action, due to unmatched genetic tools available in *S. cerevisae* 179 (Fig. 3).

By iteratively moving between simple cellular models and patient derived cells, we will be able to elucidate mechanisms and evaluate patient-specific drug targets. Ultimately, this will enable scientists to conduct more significant animal and clinical trials in various neurodegenerative diseases.

### Genetic screens

Large-scale genetic and chemical genetic approaches in yeast have provided important insight into the molecular basis of various neurodegenerative disorders. The first large-scale genetic screen used 4850 deletion yeast strains and successfully identified 86 genes enhancers of aSyn toxicity, of which 29% were involved in vesicular transport and lipid metabolism 124. Later, the same yeast collection enabled the identification of 185 modifiers of aSyn aggregation, using fluorescence microscopy 154. This study revealed that proteins involved in vesicular transport altered aSyn subcellular localization.

Using an overexpression screen, aSyn was found to block ER-to-Golgi trafficking due to the identification of enhancers and suppressors of toxicity 125. Importantly, these observations led to the identification of Rab1, the mammalian Ypt1 homolog, as a neuroprotector against dopaminergic neuron loss in animal models of PD 125.

In another genome wide-screen, using a high-expression library, Ypp1 was found to mediate the trafficking of aSyn A30P to the vacuole via the endocytic pathway, thus suppressing the toxicity of this aSyn mutant 133.

A separate yeast overexpression screen identified 40 genes that suppressed the toxicity of human WT aSyn 140. These genes were involved in ubiquitin-dependent protein catabolism, protein biosynthesis, vesicle trafficking and in the response to stress 140.

Using an integrative approach, the results from genetic screens were analyzed according to gene functionality and pathways. About ~3500 overexpression yeast strains were used and a cellular map of the proteins and genes responding to aSyn expression was obtained. Ergosterol biosynthesis and the TOR pathway were identified as modulators of aSyn cytotoxicity in yeast 128.

### Drug discovery efforts in yeast models

The events leading to protein oligomerization are likely amenable to modulation by small molecules. Thus, yeast has also been used to screen for small molecules that can reduce aSyn aggregation and toxicity.

Screening of large libraries of compounds lead to the identification of aSyn toxicity suppressors in yeast. For instance, in a large-scale screen of small molecule, ~115.000 compounds were tested for their ability to reduce aSyn toxicity, resulting in the identification of a class of structurally related 1,2,3,4-tetrahydroquinolinones 126. These compounds reduced the formation of aSyn inclusions, re-established ER-to-Golgi trafficking, and ameliorated mitochondria-associated defects induced by aSyn. The targets were further confirmed in nematode neurons and in primary rat neuronal midbrain cultures. Interestingly, these compounds also rescued rotenone toxicity in neuronal cultures, a toxin used to study mitochondrial deficits in PD 126.

The ease of manipulation makes yeast a suitable tool to explore unconventional compounds and their mechanisms of action. Mannosylglycerate, a compatible solute typical of marine microorganisms thriving in hot environments, was found to reduce aSyn aggregation in a yeast model of PD 180. Latrepirdine, a drug in phase II clinical trials, was identified as protector against aSyn by inducing its degradation through autophagy, representing a novel scaffold for discovery of robust pro-autophagic/anti-neurodegeneration compounds 181.

A novel class of molecules, cyclic peptides (CPs), was also screened in yeast 182. CPs are natural-product-like chemicals with potent bioactivity. Yeast was exploited to express a plasmid-derived self-splicing intein that liberates a CP. This approach enabled the scale-up of high-throughput screens to 10-100 times the size of a typical small molecule screen. A pool of 5 million yeast transformants were screened and two related CP constructs with the ability to reduce aSyn toxicity were identified. These cyclic peptide constructs also prevented dopaminergic neuron loss in a nematode model of PD 182.

Due to their well-defined chemical nature, small molecules are the preferred molecules used in high-throughput screenings. Nevertheless, natural compounds have emerged as attractive molecules in the context of neurodegeneration. It is largely accepted that products such as green tea, small fruits and even olive oil have, in its constitution, compounds promoting health benefits. However, the major advances regarding their targets and mechanisms of action were only achieved in the last decade. Yeast models, together with chemical and animal studies, have significantly contributed for these discoveries 175.^180^,^183^,^184^

The first small compound screen in yeast tested ~10.000 compounds and identified a group of flavonoids, quercetin and epigallocatechin gallate as protectants against aSyn toxicity in the presence of iron 185. The protection promoted by these compounds was further analyzed, and the positive effect was due to their anti-oxidant and metal-chelating activities. Importantly, (poly)phenols, and particularly quercetin and epigallocatechin gallate, have proven beneficial in cellular and animal models of PD 186,187.

The advances in biochemical tools and the assembly of multidisciplinary teams also gave a major push to the drug discovery process. In fact, the benefits of green tea have been deciphered by combining HPLC fractionation in a microplate format with screening in yeast and parallel electrospray mass spectrometry (LC-MS) 188 (Fig. 3). This integrated process enabled the rapid assessment of the efficacy of the fractions and to systematically identify their bioactive constituents. The green tea metabolites were individually examined for their pharmacological effects and, interestingly, the protective properties of *Camelia sinensis* lied on the combination of multiple catechin metabolites 188. This study emphasizes the prominence of yeast high-throughput screenings to dissect natural extracts and to explore the numerous synergistic effects of its metabolites.

The bioactivities of plant (poly)phenol extracts in the yeast aSyn model were recently investigated using viability, oxidative stress, metabolic capacity and aSyn inclusion formation as phenotypic assays 175. The most promising extract was the one from *Corema album* leaves. The dissection of the mechanism of action of this extract, focused primarily on pathways related to proteostasis, showed that it promotes autophagic flux both in yeast and in a mammalian cell model of PD 175.

Ascorbic acid, a natural antioxidant, was found to promote a significant reduction in the percentage of yeast cells bearing aSyn inclusions 189. Remarkably, this study was performed using a new microfluidic device designed to validate compounds in yeast. Additional advantages are achieved by using this device, since it offers a powerful way for studying aSyn biology with single-cell resolution and high-throughput, using genetically modified yeast cells 189.

Screenings in other disease models, as in the amyotrophic lateral sclerosis yeast model (induced by the expression of the protein TDP-43), may also provide insight into candidate compounds to be tested in aSyn yeast models. Using yeast genetics, multiple protective 8-hydroxyquinolines, natural plant alkaloids, were identified 184. Some of these compounds were also found to be protective in aSyn yeast and nematode models. The putative protective mechanisms were related to their ionophore and intracellular metal chelation activities 184. From this screening, N-aryl benzimidazole proved more potent and effective against aSyn toxicity than against TDP-43 toxicity. Thus, the yeast aSyn platform was explored to identify the mechanism of action of N-aryl benzimidazole and it was found that it reverses diverse phenotypes induced by aSyn, including the accumulation of aSyn inclusions, the generation of ROS, the block of ER-Golgi trafficking and the nitration of proteins 190. Moreover, this compound was used in an iterative yeast-to-human neuron platform 129 (Fig. 3).

Taken together, identifying novel effective disease therapies is an incredible challenge. Nevertheless, rapidly improving methodologies and iterative processes, allied with an evolving mechanistic understanding of disease, is nurturing more interdisciplinary approaches to research and fostering drug discovery, with the ultimate goal of discovering novel therapeutics for humans.

## CONCLUSIONS AND FUTURE PERSPECTIVES

The development of effective treatments and preventive therapies for PD is still a great challenge, mostly due to the scarcity of knowledge of disease-associated mechanisms that ultimately lead to neuronal dysfunction and death. As discussed herein, the versatile eukaryotic model organism *S. cerevisiae* has largely contributed to bridge this gap in PD medical research. By providing important insights into the molecular foundations of the disease as well as novel molecular targets and lead compounds with therapeutic potential. Notwithstanding, several fundamental aspects of PD pathophysiology remain to be elucidated. For example, yeast models were very helpful to clarify some facets of the still controversial role of aSyn phosphorylation, and will certainly further contribute to our understanding of other mechanisms associated with aSyn and other PD-associated genes.

Undoubtedly, research using *S. cerevisiae* as a model system enabled significant advancements in our understanding of the molecular mechanisms underlying PD. However, it should be noted that, as a simplified model system, it also has natural limitations that need to be obviated by further validations in more complex models. Indeed, iterative processes using models with different degrees of complexity have proven to be a powerful strategy to investigate the fundamental aspects of neurodegenerative diseases, thereby accelerating drug discovery.
